# DNA-directed immobilization of horseradish peroxidase onto porous SiO_2_ optical transducers

**DOI:** 10.1186/1556-276X-7-443

**Published:** 2012-08-08

**Authors:** Giorgi Shtenberg, Naama Massad-Ivanir, Sinem Engin, Michal Sharon, Ljiljana Fruk, Ester Segal

**Affiliations:** 1 The Inter-Departmental Program of Biotechnology, Technion – Israel Institute of Technology, Haifa, 32000, Israel; 2 Department of Biotechnology and Food Engineering, Technion – Israel Institute of Technology, Haifa, 32000, Israel; 3 Karlsruhe Institute of Technology, DFG – Center for Functional Nanostructures, Karlsruhe, 76131, Germany; 4 Department of Biological Chemistry, Weizmann Institute of Science, Rehovot, 76100, Israel; 5 The Russell Berrie Nanotechnology Institute, Technion – Israel Institute of Technology, Haifa, 32000, Israel

**Keywords:** Porous Si, DNA immobilization, Enzyme, Nanostructure, Biosensor

## Abstract

Multifunctional porous Si nanostructure is designed to optically monitor enzymatic activity of horseradish peroxidase. First, an oxidized PSi optical nanostructure, a Fabry-Pérot thin film, is synthesized and is used as the optical transducer element. Immobilization of the enzyme onto the nanostructure is performed through DNA-directed immobilization. Preliminary studies demonstrate high enzymatic activity levels of the immobilized horseradish peroxidase, while maintaining its specificity. The catalytic activity of the enzymes immobilized within the porous nanostructure is monitored in real time by reflective interferometric Fourier transform spectroscopy. We show that we can easily regenerate the surface for consecutive biosensing analysis by mild dehybridization conditions.

## Background

Nanostructured porous Si (PSi) has emerged as a promising material for optical biosensing applications due to its large internal surface area and tunable optical properties [1-3]. Numerous biosensing applications, including the detection of DNA hybridization [4], proteins [5,6], and enzymatic activity [7,8], have been presented, demonstrating the advantages of these nanosystems in terms of improved detection sensitivity, label-free and real-time rapid analysis. However, the major challenge in designing these biosensors arises from the intrinsic instability of the recognition element during the immobilization procedures onto the transducer’s surface [9-11].

The present work describes a highly versatile approach for reversible enzyme conjugation to a porous SiO_2_ (PSiO_2_) surface via DNA-directed immobilization (DDI). This approach has been successfully used for the immobilization of different classes of enzymes onto a range of solid supports, without affecting their biological activity [9,12]. DDI strategy utilizes the site-selectivity and the high affinity of Watson-Crick base pairing between two complimentary strands. Taking into account that DNA hybridization is a reversible process, the anchored enzyme as well as the biosensor surface can be regenerated and reused for multiple reaction cycles. Herein, we demonstrate this concept for a model enzyme, horseradish peroxidase (HRP), which is one of the most active peroxidases and often used as a powerful tool in biotechnology (e.g., organic synthesis, immune-detection, and wastewater treatment) [13,14].

## Methods

### Preparation of porous SiO_2_ nanostructures

Single side polished on the <100 > face oriented and heavily doped p-type Si wafers (0.8 to 1.0 mΩ·cm resistivity, B-doped, from Siltronix Corp., Archamps, France) are electrochemically etched in a 3:1 (*v*/*v*) solution of aqueous HF (48%, Merck, Whitehouse Station, NJ, USA) and ethanol (99.9%, Merck) at a constant current density of 385 mA cm^−2^ for 30 s. Si wafers with an exposed area of 1.33 cm^2^ are contacted on the backside with a strip of aluminum foil and mounted in a Teflon etching cell; a platinum mesh is used as the counterelectrode. After etching, the surface of the wafer is rinsed with ethanol several times and dried under a dry nitrogen gas. The freshly-etched PSi samples are thermally oxidized in a tube furnace (Thermolyne) at 800°C for 1 h in ambient air, resulting in a PSiO_2_ layer.

All materials were purchased from Sigma Aldrich Chemicals (St. Louis, MO, USA) unless mentioned otherwise.

### Preparation of enzyme-DNA conjugates

To prepare the enzyme-DNA conjugate, 100 μL of a 100-μM solution of 5′-thiol-modified single-stranded oligonucleotide (cD1 sequence) in TE buffer (10-mM Tris and 1-mM EDTA) is mixed with 60 μL 1,4-dithiothreitol (1 M) and incubated overnight at 37°C. HRP type VI (0.92 mg) is dissolved in 200 μL of 50-mM phosphate buffered saline (PBS, pH 7.4) and incubated for 1 h at 37°C with sulfo-succinimidyl-4-(N-maleimido-methyl)cyclohexane-1-carboxylate (sulfo-SMCC) obtained from Thermo Scientific Pierce Protein Biology Products, Rockford, IL USA (2 mg in 60 μL of N,N-dimethylformamide). Both the DNA and the protein reaction mixtures are purified by two consecutive gel-filtration chromatography steps using NAP5 and NAP10 columns (Pharmacia LKB Biotechnology AB, Uppsala, Sweden). The purified DNA and protein solutions are combined and incubated in the dark at room temperature for 3 h. The reaction mixture is concentrated to approximately 500 μL by ultrafiltration (Centricon 30, Millipore Co., Billerica, MA, USA), and the buffer is changed to Tris (20-mM, pH 8.3) during this step. The conjugate is purified by anion exchange chromatography on a MonoQ HR 5/5 column (Pharmacia) using linear gradient over 25 min (AKTA^TM^ purifier, Amersham Biosciences, Piscataway, NJ, USA; buffer A 20-mM Tris, pH 8.3 and buffer B 20-mM Tris and 1.5-M NaCl, pH 8.3). The final concentration is determined spectrophotometrically.

### Biofunctionalization of PSiO_2_

The PSiO_2_ films are first immersed in a solution of 3-glycidoxypropyl(trimethoxy)silane (GPTS) dissolved in toluene (90 mM) for 2 h. The samples are then extensively rinsed with toluene, ethanol, and acetone and dried under a nitrogen gas. The sequence of the capture strand is 5′-amino TCCTGTGTGAAATTGTTATACGCC-3′ (aD1). A solution of the amino-modified probe (100 μM) is applied onto the silanized PSiO_2_ surface and incubated for 2 h in a humidity chamber, followed by a post-cleaning process. Afterwards, 2.6-μM of the complimentary strand (cD1)-modifiedHRP conjugates are incubated onto the PSiO_2_ for 30 min. Finally, the PSiO_2_ samples are rinsed with 50-mM PBS, soaked in the buffer for 20 min, and vigorously rinsed again with PBS to remove any unbounded species from the surface. All processes are carried out at a room temperature (RT), below the melting temperature of the DNA (65.6°C). Mild basic conditions are used for dissociation of the hybridized complex (0.1-M NaOH for 5 min) to allow surface regeneration.

### Fluorescent labeling and fluorescence microscopy

Fluorescently labeled (5′-TAMRA-modified) complimentary strand (TAMRA-cD1, 1 μM) is used to confirm DNA hybridization onto the modified PSiO_2_ surface Following the introduction the fluorescently tagged strand, the PSiO_2_ samples are characterized using TIRF iMIC microscope, and images are taken using interline transfer CCD (Andor Clara E, Till Photonics, Munich, Germany) and fast filter-based illumination system with 488 nm TIRF laser beam or the oligochrome light. Data are analyzed by LA live acquisition software.

### Optical measurements

Interferometric reflectance spectra of PSiO_2_ samples are collected using an Ocean Optics CCD USB 4000 spectrometer (Dunedin, FL, USA) fitted with a microscope objective lens coupled to a bifurcated fiber optic cable. A tungsten light source was focused onto the center of the sample surface with a spot size approximately 1 to 2 mm [2]. Reflectivity data are recorded in the wavelength range of 400 to 1,000 nm, with a spectral acquisition time of 100 ms. Both illumination of the surface and detection of the reflected light are performed along an axis coincident with the surface normal. All the optical experiments are conducted in a fixed cell in order to assure that the sample’s reflectivity is measured at the same spot during all the measurements. All optical measurements are collected in aqueous surrounding. Spectra are collected using a CCD spectrometer and analyzed by applying fast Fourier transform, as previously described [15,16].

### Enzymatic activity of HRP

Ampliflu Red stock solutions are prepared according to the manufacturer’s instructions using 50-mM PBS buffer (pH 7.4). Final concentrations of 1-mM H_2_O_2_ and 0.1-mM Ampliflu Red are used to characterize the HRP activity levels [9]. Solutions of 200 μL are placed on the HRP-modified PSiO_2_ samples, and the reaction progress is monitored for 8 min at RT. The fluorescence values of the reaction product resorufin are recorded at 590 nm, using an excitation wavelength of 530 nm.

The relative activity is calculated by the following relationship:

(1) $$Rel.\;Activity.=\frac{Enzyme\;activity\;using\;DDI\;strategy}{Enzyme\;activity\;in\;solution}$$

### Optical sensing of HRP activity

The PSiO_2_ is washed with 50-mM PBS buffer solution (pH 7.4) for 30 min. Then, 0.8-mM of 4-chloro-1-naphthol in PBS buffer is introduced and continually cycled through the flow cell for 20 min. Finally, 0.16-M H_2_O_2_ is added to the cycled solution for HRP surface activation. The reflectivity spectra of the sample are recorded throughout the experiment.

## Results and discussion

### Biofunctionalization of PSiO_2_ with DNA-enzyme conjugates

**Figure 1:**
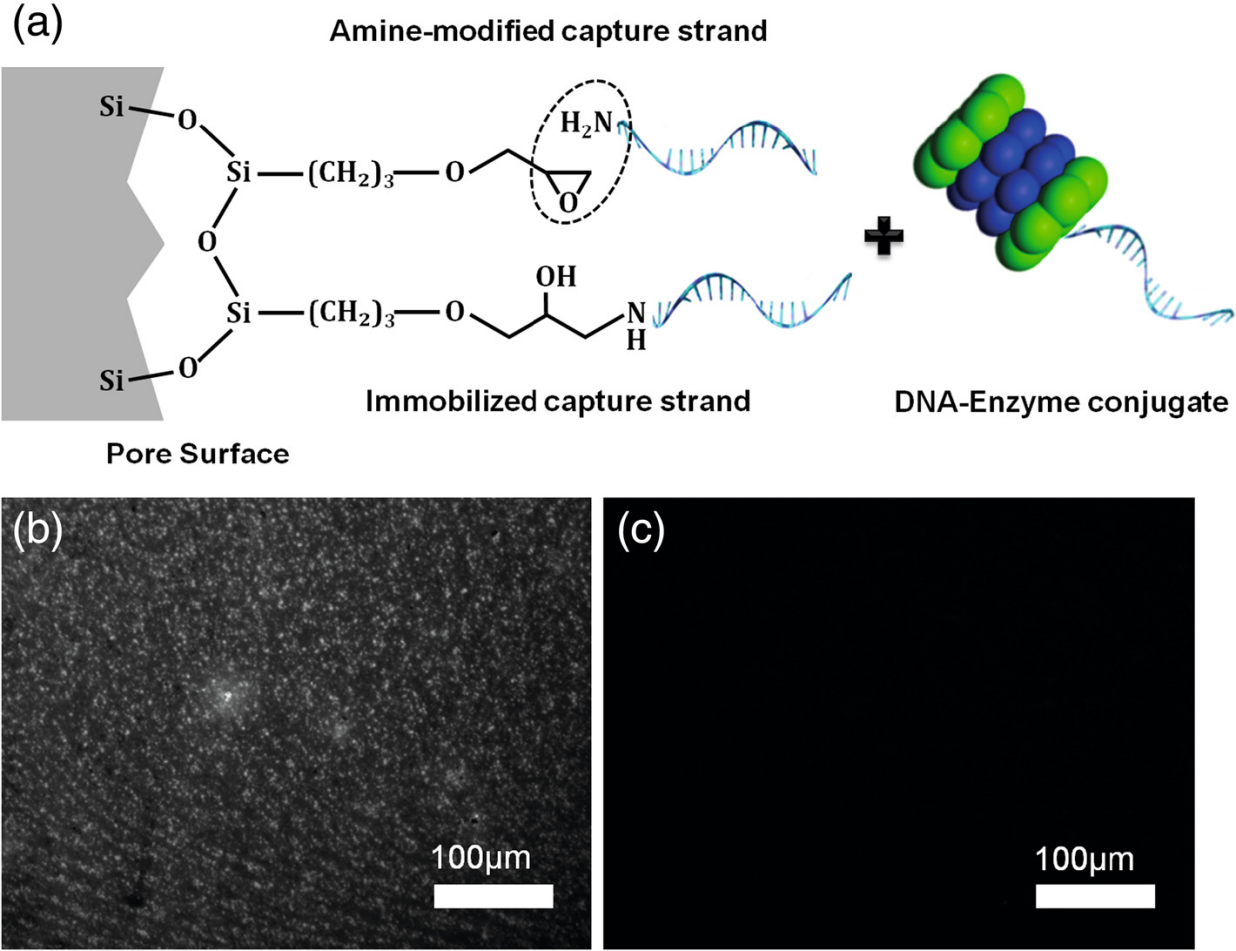
**Conjugation of enzymes to a porous SiO**_**2**_**surface using DDI approach.(a)** A schematic representation of the synthetic steps required for enzyme immobilization onto PSiO_2_ through DDI approach. The porous scaffold is first modified with GPTS to graft the amine-modified capture strand (aD1) that serves as an anchor for the following hybridization process. **(b)** Results of fluorescence labeling experiments to confirm the specificity of the DDI method. Complete bioconjugation; aD1 is attached to the silane-modified porous surface followed by hybridization of the complementary strand labeled with TAMRA fluorophore. **(c)** Control experiment; no GPTS is used (silanization step).

The PSiO_2_ surfaces are prepared from a highly doped p-type single-crystal Si wafer, polished on the <100 > face using an anodic electrochemical etch. The resulting nanostructure is then thermally oxidized to generate a stable and a more hydrophilic scaffold [17]. The structural properties are as previously described by Massad-Ivanir et al. [16]. Briefly, the porous layer is 7,880 ± 60 nm thick with interconnecting cylindrical pores ranging in diameter from 60 to 100 nm, and the calculated porosity is approximately 80%. The semisynthetic approach for anchoring DNA-protein conjugates onto the PSiO_2_ surface is based on a well-established methodology of DNA-directed immobilization to solid surfaces, e.g., gold, glass, and polymers [9,12]. The detailed synthesis is schematically outlined in Figure 1a. First, the thermally oxidized nanostructure is epoxy-silanized with GPTS to obtain an activated surface for grafting of amine-modified single-stranded capture DNA (aD1). The complimentary thiolated oligonucleotide strand (cD1) is cross-linked to the HRP using sulfo-SMCC bi-functional linker containing NHS and maleimide moieties [9]. In the final step, the resulting DNA-enzyme (HRP-cD1) conjugates are hybridized with the capture probe to enable their immobilization onto the PSiO_2_ surface. Hybridization is confirmed by studying the annealing of a fluorescently labeled complimentary DNA strand to the aD1-modified PSiO_2_. Figure 1b depicts a fluorescence microscope image of aD1-modified PSiO_2_ surface following exposure to the TAMRA-cD1, revealing a large number of small fluorescent regions on the PSiO_2_ surface. In a control experiment, the silanization step is omitted from the conjugation scheme, and the PSiO_2_ is exposed to TAMRA-cD1 only. In this case, no florescence signal is observed (Figure 1c), confirming that there is no or negligible interaction of the TAMRA-cD1 with PSiO_2_ surface in the absence of the immobilized aD1. Thus, these experiments demonstrate that the presence of the complementary capture strand is necessary for the immobilization of the TAMRA-cD1 onto the biosensing platform.

### Reflective interferometric Fourier transform spectroscopy

**Figure 2:**
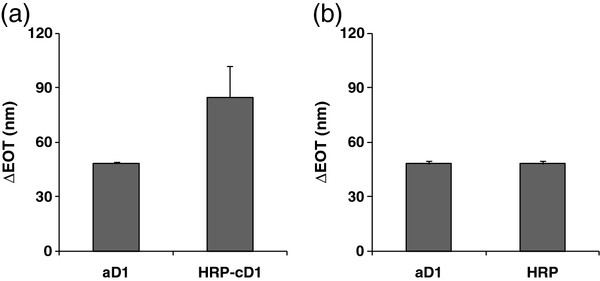
**EOT changes upon biofunctionalization of porous SiO**_**2**_**with HRP through DDI.(a)** Complete biofunctionalization process; hybridization of the complementary strand modified with HRP (HRP-cD1) to the anchored strand (aD1). **(b)** Control experiment; non-modified HRP attachment to aD1 modified surface.

Reflective interferometric Fourier transform spectroscopy (RIFTS) is used as a complimentary tool to monitor the optical changes occurring during the biofunctionalization steps onto of the PSiO_2_ surface. This method presents high sensitivity to small changes in the average refractive index of the porous thin film, allowing for direct and real-time monitoring the binding of different species to the pore walls [1,3,5,7,8]. The reflectivity spectrum of the thin PSiO_2_ film consists of a series of interference fringes that result from a Fabry-Pérot interference. This fringe pattern arises from reflections at the top and at the bottom of the film so that the measurement is made over the entire volume of the system. The effective optical thickness (EOT) refers to the 2 *nL* term in the Fabry-Pérot formula (where *n* is the average refractive index; *L*, the thickness of the film; and *λ*, the wavelength of the incident light) [15]. A change in the average refractive index leads to a shift in the observed reflectivity spectrum that correlates with EOT changes. It is expected that the chemical modification of the porous nanostructure (as previously outlined in Figure 1a) will result in a redshift of the EOT due to the increase in the average refractive index upon attachment of different species to the pore walls. It should be noted that the nanostructure is designed to allow proper infiltration of the DNA conjugates (approximate size of 10 nm [18]) into the pores, characterized by an average diameter of 80 nm. Indeed, significant EOT changes are observed after each of the described immobilization steps, see Figure 2a. Following the grafting of aD1 to the epoxy-functionalized PSiO_2_, an EOT increase of 49 ± 1 nm is detected. DDI of the HRP-cD1 conjugates (230 ng in 2 μL volume) causes an additional EOT increase of 85 ± 17 nm. Additionally, the specificity of the hybridization is validated by addition of non-modified HRP to the aD1-modified PSiO_2_, resulting in insignificant EOT changes (Figure 2b). Thus, the RIFTS results further confirm that the enzyme immobilization onto the PSiO_2_ surface occurs through Watson-Crick base pairing.

### Enzymatic activity assays

**Figure 3:**
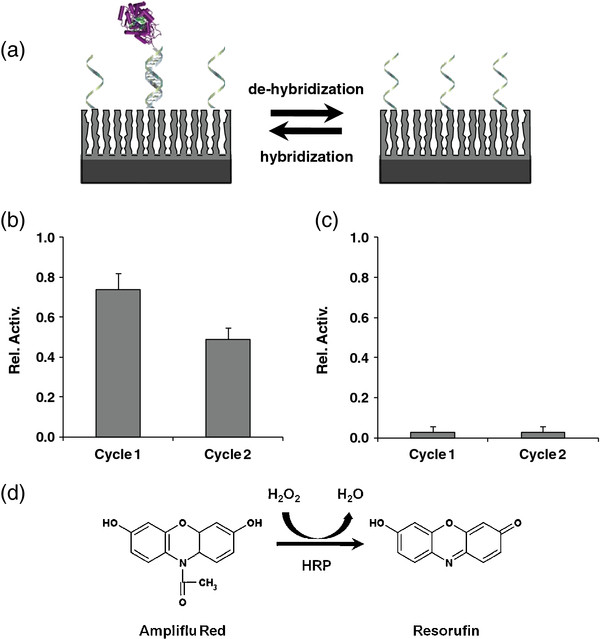
**Reversible HRP immobilization onto porous SiO**_**2**_**surface using DDI and the corresponding enzymatic activity.(a)** A schematic illustration of reversible enzyme immobilization onto PSiO_2_ surface using DDI approach. Note: These schematics are for illustration purposes only as all modifications occur also inside the pores. **(b)** Relative activity of HRP immobilized via DDI onto PSiO_2_. **(c)** Removal of the DNA-enzyme conjugates form the surface by mild dehybridization. Two consecutive cycles of DNA-enzyme hybridization/dehybridization are performed. **(d)** HRP enzymatic activity is quantified using the Ampliflu Red assay in which oxidation of the non-fluorescence Ampliflu substrate occurs in the presence of HRP and H_2_O_2_ to fluorescence resorufin.

The enzymatic activity of the anchored enzymes onto the porous scaffold is studied by monitoring the oxidation products of HRP (Figure 3d). The modified PSiO_2_ samples are exposed to a substrate solution of Ampliflu Red in PBS, and the collected solution is spectrophotometrically analyzed. Figure 3b depicts the high relative activity (74%) of the immobilized conjugates compared to non-modified HRP activity in a solution (Equation 1). Thus, the modified enzymes retain their specificity and activity once anchored and confined on the porous support. An important advantage of the DDI anchoring is the ability to regenerate the surface, as schematically shown in Figure 3a, by applying mild dehybridization conditions, e.g., pH change or temperature increase (above the melting temperature of the DNA pair). Indeed, following a mild-base wash, an insignificant relative enzymatic activity value of less than 3% is attained (Figure 3c), indicating that the PSiO_2_ surface can be regenerated. In addition, a consecutive cycle of hybridization/dehybridization is carried out to demonstrate the reversibility of DDI method for the characterization of small aliquots (2 μL) of the recovered enzyme and its reuse. These experiments show the high reversibility and reproducibility of the immobilization strategy as relative activity values of 49% and less than 3% are recorded, followed by DDI and dehybridization processes, respectively (Figure 3b,c).

### Optical detection of enzymatic activity

**Figure 4:**
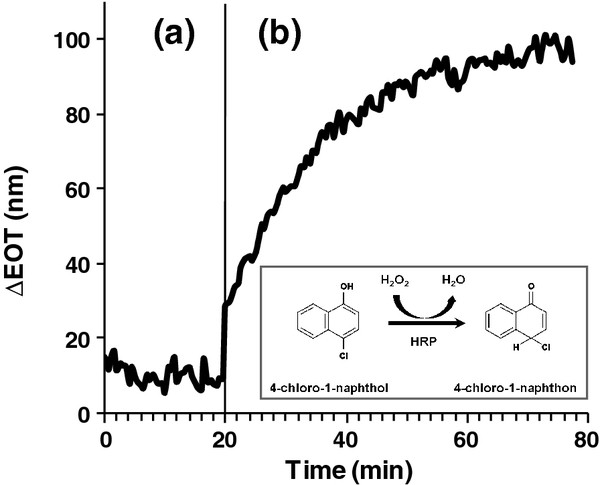
**Optical response of HRP-modified porous SiO**_**2**_**to enzymatic reaction products infiltrating into the nanostructure.** The HRP-modified PSiO_2_ nanostructure is pretreated with 50-mM PBS (pH 7.4) to minimize nonspecific adsorption of proteins. **(a)** Wash with 0.8-mM 4-chloro-1-naphthol in PBS buffer (pH 7.4). **(b)** Addition of H_2_O_2_ to the cycled solution. The PSiO_2_ biosensor is fixed in a custom-made flowcell, and the reflectivity spectra are recorded every 30 s. Inset: oxidation of 4-chloro-1-naphthol in the presence of HRP and H_2_O_2_.

Previous sections have demonstrated that the DNA-HRP conjugates remain catalytically active after the DDI onto the PSiO_2_ nanostructure. Next, we investigated the potential of this platform to act as an optical biosensor for monitoring enzymatic activity through RIFTS experiments. Thus, HRP-modified PSiO_2_ is fixed in a flow cell setup and is exposed to various substrate/buffer combinations. The reactions are monitored in real time by acquisition of the reflectivity spectra of the porous thin film. The recorded EOT values prove a direct measure of the amount of enzymatic activity products infiltrating the pores, as previously shown by Orosco et al. [7]. Figure 4 depicts the EOT change of the HRP-modified nanostructure following the introduction of 4-chloro-1-naphthol, which oxidizes in the presence of peroxidases into insoluble 4-chloro-1-naphton [19] (Figure 4, inset). A rapid increase of 90 nm in the EOT value is observed after injection of 0.16-M H_2_O_2_ to the cycled solution (Figure 4b). The EOT increase is attributed to infiltration and accumulation of the oxidation products in the pores as the enzymatic reaction occurs [20]. Control experiments with bare and only aD1-modified PSiO_2_ (no immobilized enzyme) exposed to the same conditions exhibit insignificant changes in EOT (data not shown), indicating that the biosensor platform is indeed specific for monitoring the enzymatic activity products.

## Conclusions

We demonstrate that DNA-directed protein immobilization is an elegant and facile method for specific and reversible anchoring of a model enzyme, i.e., HRP onto PSiO_2_ Fabry-Pérot thin films. HRP specific immobilization through Watson-Crick base pairing onto the porous nanostructure is confirmed by both fluorescence microscopy and RIFTS. The surface-immobilized HRP retains relatively high enzymatic activity in comparison to its activity in solution. We show that the catalytic activity of the HRP immobilized within the PSiO_2_ thin film can be monitored in real time by RIFTS technique. For biosensor design, the DDI method allows the use of small enzyme quantities for monitoring the reaction while allowing both enzyme and surface to be regenerated for subsequent usage. This ‘proof-of-concept’ biosensor scheme can be potentially extended for systematic analysis of the enzyme of interest under unlimited experimental setups.

## Competing interests

The authors declare that they have no competing interests.

## Authors’ contributions

GS carried out PSi synthesis, enzyme immobilization, enzyme activity assays, biosensing experiments, and data analysis. He also wrote the manuscript. NMI contributed to the conception of the experiments and was involved in the synthesis and characterization of PSi. SE synthesized the DNA-enzyme conjugates. MS participated in the design of the study. LF conceived the study and coordinated the synthesis and characterization of DNA-enzyme conjugates. ES conceived and coordinated the study, participated in the design of the study and data analysis, and drafted the manuscript. All authors read and approved the final manuscript.
